# The American and Colonial Journals

**Published:** 1841-10

**Authors:** 


### II. THE AMERICAN AND COLONIAL JOURNALS.

### *Origin of the Operation for Strabismus.*

[It would appear from the following communication from S. Y. Atwell, Esq. of Providence, to Dr. Rivers of the same city, that an American Physician, and not Dr. Stromeyer, has the merit of having first suggested this operation:]

In the years 1812-13, I attended courses of surgical and anatomical lectures delivered before the Medical School of Brown University, by William Ingalls, M.D., of Boston, then the Professor of Anatomy and Surgery in that institution: being subject myself to this infirmity (strabismus), Dr. Ingalls took frequent opportunities to explain to me the method of its surgical cure; he did this by dissection of the eye itself, explanation of the power and disposition of the several muscles appertaining to that organ, and showed me how, by a division of one or more of them, the eye might be brought to its proper place. In my own case I know he proposed to divide the *rectus internus*. So strongly was I impressed with the practicability and success of this operation, that I strongly urged my father to permit me to submit to the operation; but upon the nature of the operation being explained to him, he declined the permission, because he feared the effect might be to turn the eye the other way.

*Medical Examiner. Feb.* 20, 1841.

### *Cases of Poisoning by Arsenic—Treated by the Hydrated Peroxide of Iron.* By T. T. Smiley, M.D., and J. M. Wallace, M.D.

On the 3d day of January, 1840, the family of Mr. Constant Gigon, residing at No. 148, Pine Street, Philadelphia, was poisoned by eating a pudding made of Indian meal, in which arsenic had been mixed, probably for the purpose of destroying rats. The precise quantity of arsenic contained in the pudding could not be ascertained; but, judging by the effect produced, must have been very great. The whole family, consisting of eight persons, was affected with symptoms more or less violent. An hour after the pudding had been eaten, on finding a general sickness pervading the family, a dose of ipecacuanha had been administered to each of the individuals affected, by Mr. Zollickoffer, an apothecary residing in the neighbourhood.

On the arrival of Dr. Smiley, who was first called in, poisoning from arsenic was at once suspected,—and on that supposition copious draughts of warm water, with sweet oil, were administered, and followed by whites of eggs in large quantities. During the operation of the foregoing remedies, given with a view to evacuate the stomach, and remove the poison as thoroughly as possible, Mr. Zollickoffer was requested to prepare a large quantity of the hydrated peroxide of iron, for the purpose of being administered as an antidote to that portion of arsenic which always in such cases remains in the stomach, and cannot be entirely evacuated by vomiting. As soon as the hydrated peroxide of iron could be prepared, it was administered in tablespoonful doses, at short intervals, to all the patients, and as often as rejected by vomiting, was immediately repeated. In nearly all the patients an almost entire subsidence of the most violent symptoms soon followed, and the burning sensation of the œsophagus and stomach greatly diminished. Two of the patients died.

In the two cases which proved fatal, death appears to have ensued from the direct effects of the poison on the nervous system. It might even be doubted whether the injury sustained by the stomach in either case was sufficient to have produced death by the subsequent inflammatory action, and possibly both these patients might have recovered, provided the direct shock to the system could have been obviated, and reaction produced.

It is worthy of observation that every one of the patients who were able to retain the hydrated peroxide of iron on the stomach speedily recovered, and were restored to perfect health, with the entire exemption from any symptom indicating the existence of chronic imflammation of the stomach which has heretofore so uniformly followed poisoning from arsenic in the few cases which have not proved immediately fatal.

The great and almost immediate alleviation of the urgent symptoms which followed in every instance in which the hydrated peroxide of iron could be retained on the stomach, affords the strongest evidence that the beneficial results ascribed to that remedy in such cases have not been overrated. Fortunately, few instances have occurred in which the virtue of that antidote could be tested on so large a scale.

NOTE.—The contents of the stomachs of the two patients who died were submitted to a careful chemical examination by Dr. Rogers, and the existence of arsenic in them placed beyond a doubt. A large quantity of metallic arsenic was produced both from the contents of the stomachs and the remains of the pudding.

*Medical Examiner. October* 24, 1841.

---

*Anomalous Case of Periodical Suspended Perception.*
By JOSEPH JAMES RIDLEY, M.D.

[THIS seems a case of catalepsy, of which the most curious feature is the loudness of the eructations. Were not the case authenticated as it is, it would seem fabulous : but as the French proverb has it, truth is not always truthlike.]

Harry, a man servant of Mrs. W., of N. C., was from his youth of a strong and vigorous constitution, plethoric habit, and in the intervals of his paroxysms of good general health. In about his thirty-fifth year, Dr. R. was requested to see him in a paroxysm simulating apoplexy. The remedies for apoplexy were used in vain. He lay perfectly torpid and insensible; the symptoms became hourly more threatening until recourse was had to cleansing the primæ viæ. The administration of a laxative restored him to sensation, and with the restoration of his perceptions all symptoms of disease disappeared. In a few days he had a second attack; the symptoms as before. The same treatment relieved him. His mistress, not famed for abundance of the "milk of human kindness," suspected his honesty, and cruelly scourged and burnt him in his paroxysms without making any impression upon his perceptions. His paroxysms periodically returned, and became more frequent gradually. He was likely to prove an expensive piece of property ; and his mistress, resolved to incur no more expense, sold him at a reduced price to Dr. R., who, upon taking him home, essayed every power of the medical art to ascertain and remove the disease. He discovered that in his paroxysms the abdomen was uniformly extremely tense ; the colon was, to all appearance, the seat of the disease. Upon making use of friction upon the abdomen, a vast quantity of flatus was eructated, and the urgent symptoms gradually gave way. After discharging an inconceivable quantity of gas, his senses returned; his paroxysms gradually became quotidian. Dr. R. was forced to take him with him whenever he was called from home, about the periodical recurrence of the paroxysm. The longer the paroxysm continued, the more difficult was it to restore him to sensibility. A most remarkable fact was the circumscribed spot upon which friction afforded relief; a five franc piece would cover the space on which it was necessary to rub ; friction on any other part of the abdomen was certain to aggravate the symptoms fourfold. This spot was, I think, about four or six lines to the right of the umbilicus.

Another remarkable fact connected with this case was the incredible force of his eructations: when properly rubbed, the flatus would escape with such force *as to be heard one or two miles*[!] I am aware, Messrs. Editors, that this seems rather a Munchausen story ; it transcends the bounds of credibility, I know. It is difficult to believe a statement conflicting so palpably with the results of our observation. The lungs had no part in the production of the report ; it was exclusively from the stomach and intestines. The statement, in its general outline, would be avouched by a whole neighbourhood, and several physicians, to whom the case was one of inexplicable difficulty and interest. When the paroxysm was coming on, before the power of locomotion was suspended, he would wander about, regardless of those things that affect the senses. Upon one occasion he was in the act of jumping into a well, and was most narrowly and opportunely rescued. Upon the return of sensibility, he was like a man suddenly aroused from sleep, before his perceptions are fully brought into exercise.

The disease lasted several years and finally exhausted itself.

*Medical Examiner. January* 9, 1841.

---

*On the Presence of Kiesteine in the Urine as a Test of Pregnancy.* By W. M. M'PHERTERS and J. C. PERRY, Resident Physicians at Philadelphia Hospital.

WE have instituted a series of experiments for the purpose of ascertaining the existence of kiesteïne in the urine of pregnant women. They have been conducted after the manner pursued by Dr. Golding Bird, as published in Guy's Hospital Reports, of April, 1840. Daily observations were made, and every change as it presented itself carefully noted. The results of our observations are as follows: The urine of twenty-four out of twenty-seven women, in different stages of pregnancy, from the second to the ninth month of utero-gestation, on the second day began to lose its transparency; on the third, became quite opalescent. From the second to the fourth day, a whitish scum made its appearance, which, as described by previous observers, might aptly be compared "to the layer of greasy matter which covers the surface of fat broth when it has been allowed to cool." This continued to increase until the fifth or sixth day, when, in each case, it was so unequivocally marked as not to be mistaken. From this until the fourteenth day, when the observations closed, the pellicle became gradually thinner by the detachment and subsidence of flocculi, though it never entirely disappeared. The odour, which was peculiar, could not be recognized by us as "cheesy," although our attention was particularly directed to this point. The filtered specimens of the same urine underwent similar changes.

Of the three remaining cases, in which the pellicle did not appear, one was labouring under peritonitis, and subsequently had an attack of puerperal fever, and another had incipient phthisis. We are unable to say whether the third was labouring under disease or not.

The urine of one suckling woman, two weeks after delivery, presented all the characteristics of kiesteïne. That of two others, twelve months after delivery, whose children were still at the breast, underwent no change.

So far as we observed, the presence or absence of milk in the breasts, previous to delivery, appeared to exercise no influence over the formation of the scum.

An elevated temperature of from 60° to 80° appears most favorable to the speedy and perfect formation of the kiesteïnic pellicle, whilst one low enough to freeze the urine occasionally prevents or very materially retards the process. This may perhaps account for some apparent discrepancies between our observations and those of Dr. Bird, as some of our experiments were conducted at rather a low temperature.

By way of instituting a comparison at the same time, the urine of twenty-seven unimpregnated women was submitted to examination, but underwent no changes indicative of kiesteïne. Of these, twelve were regular and healthy, nine were labouring under gonorrhœa, and were generally irregular. Three had chronic leucorrhœa, two indolent ulcers on the leg, and one was in the last stage of phthisis. The six last mentioned also had amenorrhœa of long standing.

The urine of four healthy men, and of two labouring under catarrh of the bladder, underwent no change.

From the nature of our situation, we had an opportunity of tracing the history of each case examined. The urine, in every instance, was that first voided in the morning, and that of no one was taken who did not remain long enough in the house either to be delivered or to place their state beyond a doubt.

From what has hitherto been published on this subject, as well as from the further observations made by ourselves, it will be seen that this matter is worthy of further investigation; and that whilst it cannot be relied on as an absolute test of pregnancy, yet when taken in conjunction with other symptoms, it forms a valuable aid to diagnosis.

*American Medical Library and Intelligencer. March* 15, 1841.

---

*On the Use of the Chloride of Silver.* By J. C. PERRY, M.D. one of the Resident Physicians of the Philadelphia Hospital.

IT must be universally acknowledged, that the nitrate of silver administered by the mouth can never act as nitrate of silver on the system, since any dose which could be ventured upon must, immediately on entering the stomach, be converted into the chloride of silver, from the chloride of sodium of our food, or the hydrochloric acid of the gastric juice.

The chloride must then, à priori, be considered as efficacious equally with the nitrate, while it will be found less uncertain in its effects, more convenient for exhibition, less liable to decomposition, and free from its nauseous taste. It may be given, too, in any dose thought necessary to produce the alterative and tonic action of silver without danger.

In less doses than thirty grains no irritating or manifest effects result. Thirty grains given at once will generally produce emesis. The best form of exhibition is in pill. To children it can be given as a powder suspended in syrup.

Twelve grains administered daily for three months have produced no unpleasant symptoms, and none of my numerous and long-continued courses of the remedy have been followed by discoloration of the skin.

In epilepsy, three grains, given four or five times daily, produced effects similar to those of nitrate of silver, but more marked.

In chronic dysentery, half a grain to three grains thrice daily produces immediate diminution in the number of stools, and tormina, with amelioration in the character of the stools and other symptoms.

I have not ventured to use it freely in acute dysentery, but in a few cases in which it has been administered, its effects seemed equally beneficial.

In chronic and colliquative diarrhœa, benefit was derived from its use in the same doses, but not so markedly, uniformly, or permanently.

The catamenia suspended for years have, without any adjuvant treatment, returned after its free exhibition for two or three weeks.

One case of secondary syphilis (the only one in which it has been tried) improved rapidly under it.

*American Medical Library and Intelligencer. February,* 1841.

---

